# Higher revision and secondary surgery rates after ACL reconstruction in athletes under 16 compared to those over 16: a case-control study

**DOI:** 10.1186/s13018-025-05935-5

**Published:** 2025-06-17

**Authors:** Miklós Tátrai, Tamás Halasi, András Tállay, Annamária Tátrai, Atilla Ferenc Karácsony, Eszter Papp, Attila Pavlik

**Affiliations:** 1 Doctoral Office, Semmelweis University, Üllői street 26, Budapest, H-1085 Hungary; 2 Buda Health Center, Királyhágó Street 1-3, Budapest, H-1126 Hungary; 3 Faculty of Sport Medicina, Semmelweis University, Gaál József Street 9-11, Budapest, H-1122 Hungary; 4 Faculty, of Social Sciences, Eötvös Lóránd University, Pázmány Péter promenade 1/A, Budapest, H-1117 Hungary; 5 Buda Hospital of the Hospitaller Order of Saint John of God, Frankel Leo Street 17-19, Budapest, H-1027 Hungary; 6 National Institue for Sports Medicine, Karolina Street 27, Budapest, H-1113 Hungary

**Keywords:** ACL, Adolescent, Revision surgery, Female, Secondary surgeries

## Abstract

**Background:**

The incidence of anterior cruciate ligament (ACL) reconstructions among adolescents, particularly those involved in high-risk sports, has increased. Despite surgical advancements, outcomes remain worse than in adults. This study aimed to assess ACL reconstruction outcomes in patients under 16 years and compared to older patients. The prevalence of high pivoting sports in those undergoing revision or contralateral ACL reconstruction was also analyzed. We hypothesized that younger athletes experience poorer outcomes and higher rates of secondary surgeries.

**Methods:**

This study evaluated ACL reconstruction outcomes in patients under 16 years (Group 1) and compared them with those over 16 years (Group 2). A retrospective analysis was conducted on patients who underwent primary arthroscopic ACL reconstruction between 2007 and 2022. Ipsilateral and contralateral surgeries were analyzed in both groups. Patient-reported outcomes (Lysholm score, Knee Injury and Osteoarthritis Outcome Score [KOOS], Tegner Activity Scale, and International Knee Documentation Committee [IKDC] scores) were compared between the two groups. The impact of sports activity level and sex on revision rates was examined. Statistical tests, including two-sample Z tests and two-sample t-tests, were used for analysis. Secondary surgeries were defined as additional procedures after ACL reconstruction, microfracture, hardware removal, and arthrolysis.

**Results:**

Group 1 (average age: 15.2 years) included 70 patients with a follow-up of 6.9 years, and Group 2 (average age: 30.8 years) included 87 patients with a follow-up of 3.66 years. A significant age difference was found (*p*<0.001). Group 1 had higher rates of contralateral ACL surgeries (18.3% vs. 1.1%, *p*=0.03), meniscus surgeries (26% vs. 4.6%, *p*=0.003), and secondary surgeries (44% vs. 21%, *p*=0.01) compared to Group 2. Female athletes under 16 years had a significantly higher rate of contralateral ACL reconstruction (92% vs. 69%, *p*=0.020). In Group 1, the KOOS Pain score was significantly higher (95.6 vs. 94.0, *p*=0.033), but the Symptoms score was significantly lower (75.6 vs. 85.0, *p*<0.005).

**Conclusion:**

Patients under 16 years undergoing ACL reconstruction had higher rates of both contralateral and ipsilateral ACL surgeries, as well as secondary surgeries, compared to older patients. Female adolescents had a significantly higher incidence of contralateral ACL reconstruction.

Anterior cruciate ligament (ACL) ruptures are common severe injuries in athletes, with increasing incidence in both adults and adolescents [1–3]. The ACL rupture rate per 1,000 hours ranges between 0.005 and 0.2 across different team sports and it is particularly common in team ball-sports [1, 4–8]. Conservative treatment often results in poor outcomes, such as osteoarthritis, meniscus damage, and poor patient-reported outcome scores [9–11]. For this reason, surgical treatment is definitely recommended, despite the graft rupture rate being high, ranging between 5% and 13% in the literature after ACL reconstruction [12–15]. Many studies have shown that ACL reconstruction tends to have worse outcomes in young patients under 25, with a higher secondary injury rate, particularly among those returning to sport [12–19].

The purpose of this study was to assess the results of ACL reconstruction surgery in patients under 16 years of age and to compare them with data from patients older than 16 years. We also analyzed the prevalence of high pivoting sports among athletes who underwent revision and contralateral ACL reconstruction.

We hypothesized that ACL reconstruction among athletes under 16 years of age results in fewer good outcomes with a higher rate of secondary surgeries, and a greater number of high pivoting athletes who underwent revision or contralateral ACL reconstruction.

## Materials and methods

In our retrospective case-control study, we created two groups. In the first group, the patients underwent ACL reconstruction under the age of 16 years (Group 1); in the second group, the patients were older than 16 years at the time of ACL reconstruction (Group 2). The exclusion criteria were (1) multiple-ligament injuries and (2) ICRS grade III-IV osteoarthritis at the time of ACL reconstruction. Concomitant meniscal injury was not an exclusion criterion.

As the first step, the patients completed our self-completion questionnaire, in which we assessed the value of the and International Knee Documentation Committee [IKDC] subjective, Lysholm, Knee Injury and Osteoarthritis Outcome Score [KOOS], and Tegner scores, and we recorded all knee surgeries of the patients since their first ACL reconstruction. During the physical examination, we measured the Range of Motion (ROM); carried out Lachman, anterior drawer and pivot shift tests; and assessed laxity with a KT-1000 arthrometer at 67 and 89 N on both knees. The tests were carried out by two separate orthopedic surgeons. In the case of different measurements, a joint measurement was made.

In the second group (Group 2), we analyzed the Lysholm score, KOOS, Tegner Activity Scale, IKDC scores, as well as the number and types of knee surgeries.

For comparability, the data were standardized for the follow-up time to 4 years to match it to the follow-up time of the second group (Group 2).

Patients in both groups were contacted twice by telephone to take part in the study.

### Operation techniques

In patients who underwent ACL reconstruction with hamstring tendons, quadrupled semitendinosus and gracilis tendon grafts were used with an Endobutton suspensory fixation system at the femoral and two spiked staples at the tibial side. The operation technique was precisely described by Kawaguchi et al. [20].

The remaining patients underwent ACL reconstruction with a bone‒patellar tendon‒bone graft. We used an implant-free, double press-fit fixation technique for graft fixation [21, 22]. In surgeries performed with both grafts, the femoral tunnel was created via the transtibial technique. In the case of open growth zones, whenever significant growth was still expected and no other associated injuries were present, we tried to postpone the ACL reconstruction until the onset of closure. If the ACL reconstruction was performed near an open growth zone, then a fixation type bridging the zone (such as an interference screw) was not used. We used a patellar tendon graft when the growth plates had already closed.

#### Rehabilitation

The patients had the same rehabilitation protocol. The operated knee was fixed at 0° in a postoperative brace. However, during daily physiotherapy sessions, the brace was removed and the knee was mobilized within a 0–40° range to gradually restore range of motion. We initiated isometric muscle strengthening exercises immediately after the surgery. In the first week, full non-weight bearing was applied. We started a full range of movement at the 4^th^ postoperative week. The patients could begin cycling at six weeks, swimming at ten weeks, and running in a straight line at twelve weeks after the operation. Sport-specific exercises were started at week 16. We allowed the patients to return to sports within the ninth postoperative month.

#### Statistical analysis

We compared the number of surgeries, the types and levels of sports, as well as the sex distribution via the two-sample Z test, whereas the analysis of patient-reported outcomes was carried out via two-sample t tests. The level of significance was set at *p*<0.05, and the analysis was performed in R software (version: 4.2.2).

## Results

**Fig. 1:**
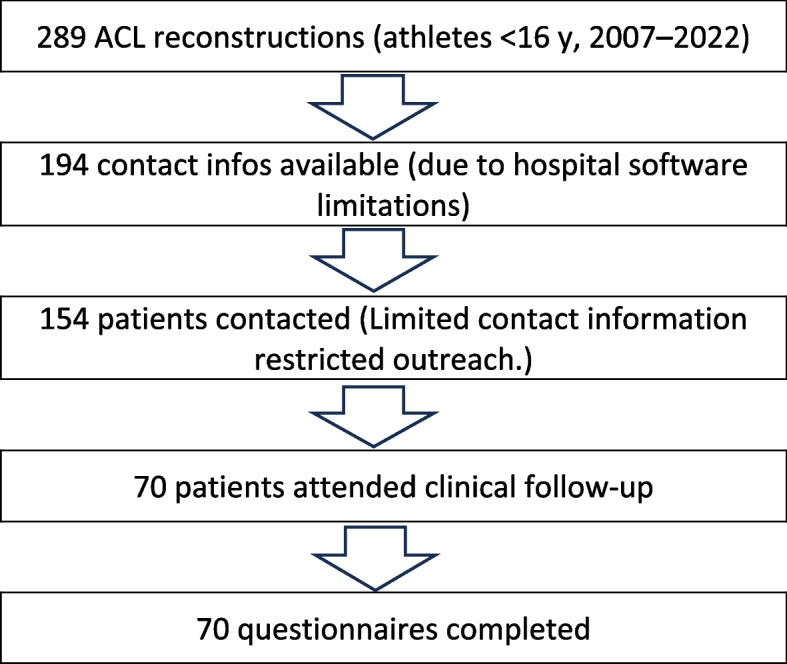
Patient selection flowchart

There were 289 ACL reconstructions among athletes under 16 years of age (Group 1) between 2007 and 2022 in our Department of Sports Surgery. Owing to multiple changes in hospital software, we only had 194 telephone numbers or email addresses. Since the patients were minors at the time of ACL reconstruction, we only had access to their parents' contact information. In several cases, the patients’ contact details were not provided, so we were only able to reach out to 154 patients. In total, we were able to include 70 patients (45%) who underwent clinical examinations during the follow-up. Due to the characteristics of the Hungarian healthcare system, the doctor–patient relationship tends to be particularly strong in cases requiring surgical intervention. Patients also demonstrate a strong attachment to the institutions where they received their treatment and surgery. Although in many cases the follow-up contact was not made directly by the operating surgeon, the patients generally responded positively to the outreach, largely due to their trust in the institution and satisfaction with their treatment. Furthermore, patients with less favorable surgical outcomes were also motivated to participate in the follow-up examinations, as this provided an opportunity for them to receive updated information regarding the condition of their knee joint. All 70 patients included completed the questionnaire in full. Figure 1.

There were 22 males and 48 females (M/F:22/48; 31%/69%) with a mean chronological age of 15.2 (13.2–15.99 years) years. Sixty-five patients underwent ACL reconstruction with hamstring grafts (semitendinosus and gracilis), and 5 patients underwent patellar tendon grafts. The mean follow-up time was 6.9 years (range: 2–19.3 years), and the mean age at the time of follow-up was 22.1 years (range: 15.2–35.2 years).

We found an improvement in the number of ACL surgeries per year in patients under 16 years of age. The number of operations rose significantly, from 5–6 procedures in the early 2000s to 2025 procedures in the late 2010s (Table 1).

**Table 1:**
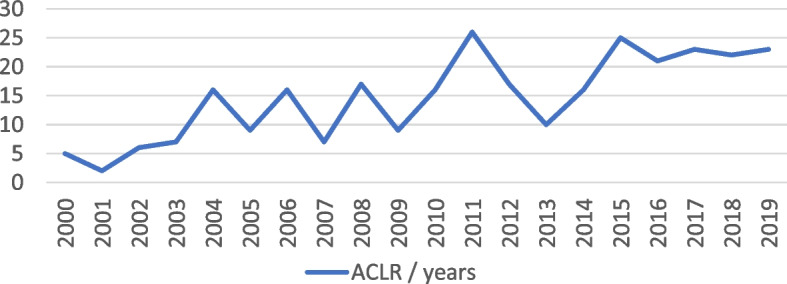
ACLR numbers between 2000 and 2020

Among patients under the age of 16 years, 10 patients underwent revision ACL reconstruction (14.1%), and 13 patients underwent ACL reconstruction on the contralateral side (18.3%). The median time of the revision ACL reconstruction was 28 months (7–66 months), whereas it was 53 months (12–138 months) for the contralateral side. Among the revisions, 70% occurred in the first three years after ACL reconstruction. Three patients (4%) underwent both revision and contralateral ACL reconstruction. A total of 28 meniscal surgeries were required in 22 patients (39.4% of the surgeries, representing 31% of the patients). In three cases, it was necessary to remove the staples (4%). There were only 31 patients (43.6%) who did not have any secondary surgeries.

Fifty-three patients (75%) had an average side-to-side difference of 1.9 mm with the KT-1000 arthrometer at 89 N. With respect to ROM, 19 patients (27%) had an average of 4 degrees less extension, whereas 36 patients (51%) had an average of 9 degrees less flexion.

The patient-reported outcome results were as follows: mean KOOS score 86.7 (48.4–100); IKDC subjective score 87.1 (54–100); and Lysholm score 89.5 (43–100). The results of the KOOS subgroup were as follows: KOOS Pain: 96.0 (75–100); KOOS symptoms: 76.5 (50–100); KOOS Daily: 98.9 (86.8–100); KOOS Sport: 88.4 (10–100); and KOOS QOL: 73.3 (6.25–100). The median Tegner score was 8.0 before and 7.0 after ACL reconstruction.

### Types of sports

Of all participants, 74% were athletes in either handball (22 athletes, 31.4%), soccer (17 athletes, 24.3%), or basketball (13 athletes, 18.6%). For simplicity, these sports are grouped under the abbreviation HSB (handball, soccer, basketball). Additionally, one athlete from each of the following sports participated: alpine skiing, acrobatics, riding, fencing, triathlon, orienteering, water polo, dancing, badminton, artistic gymnastics, athletics, karate, and volleyball (Table 2).

**Table 2:** Demographic data of the Groups

Groups	Group1	Group2	
Number	70	86	
Sex (F/M)	48/22	33/53	
Age (Years)	15,2	30,8	
Follow-up (years)	6,9	3,66	
Level of sports (C/R)	60/10	35/48	
No sport		3	
Type of Sports:			
handball	22	31	soccer
soccer	17	11	handball
basketball	14	7	alpine skiing, bicycle
Volleyball	3	3	basketball, athletics, artistic gymnastics
athletics, karate/combat sport	2	5	dancing
alpine skiing, fencing,water polo, triatlon, orienteering, dancing, riding, badminton, artistic gymnastix, acrobatics	1	2	volleyball, karate/combat sport, ice hockey, wrestling
		1	badminton, aerobic, rowing, snowboard, auto-motor sports

Legend: Demographic and sports characteristics of two groups, including age, follow-up time, sex distribution, sports participation level, and types of sports practiced

The percentage of revision was 15% among the three dominant sport types, i.e., team athletes, whereas the revision rate was only 11%-among athletes of other sports. The difference was not statistically significant (*P*=0,326). A similar pattern was observed in contralateral ACL reconstructions, with a rate of 21% in team athletes compared to 11% in others, although this difference was also not statistically significant (*P* = 0.173) (Table 3)

**Table 3:** Analysis of Revision and Contralateral Anterior Cruciate Ligament Reconstruction (ACLR) Rates According to Sport Type, Activity Level, and Sex

Type of Sport	**Revision ACLR Numbers**			**Contralateral ACLR**		
	Revision ACLR – No	Revision ACLR – Yes	Total	Revision ACLR – No	Revision ACLR – Yes	Total
Other	16(89%)	2(11%)	18(100%)	16(89%)	2(11%)	18(100%)
HSB	44(85%)	8(15%)	52(100%)	41(79%)	11(21%)	52(100%)
Total	60	10	70	57	13	70
		*P*=0,326			*P*=0,173	
Level of Sports	Revision ACLR – No	Revision ACLR – Yes	Total	Revision ACLR – No	Revision ACLR – Yes	
Recreational	9(90%)	1(10%)	10(100%)	16(89%)	2(11%)	18(100%)
Competitive	51(85%)	9(15%)	60(100%)	49(82%)	11(18%)	60(100%)
Total	60	10	70	57	13	70
		*P*= 0.338			*P*= 0.236	
Sex	Revision ACLR – No	Revision ACLR – Yes	Total	Revision ACLR – No	Revision ACLR – Yes	Total
Male	20(91%)	2(9%)	22(100%)	21(95%)	1(5%)	22(100%)
Female	40(83%)	8(17%)	48(100%)	36(75%)	12(25%)	48(100%)
Total	60	10	70	60	10	70
		*P*=0.200			*P*=0.020	

Legend: This table presents the distribution of patients who underwent revision ACLR and contralateral ACLR based on type of sport, level of sports activity, and sex

Revision ACLR – No / Yes: Indicates the number and percentage of patients who did not (No) or did (Yes) undergo revision ACL reconstruction. Contralateral ACLR – No / Yes: Indicates the number and percentage of patients who did not (No) or did (Yes) undergo ACL reconstruction on the opposite knee. Other: Refers to patients participating in sports other than Handball, Soccer, Basketball (HSB) sports. HSB: Handball, Soccer, Basketball players. Recreational: Patients who play sports casually or for leisure. Competitive: Patients who participate in organized sports at a competitive level. Male / Female: Biological sex of the patients.

Each subgroup shows the number of patients and the percentage (in parentheses) relative to the total in that subgroup. P-values are provided to indicate statistical significance of differences between subgroups. A P-value less than 0.05 is considered statistically significant.

### Level of sports

Most of the athletes played sports at a high level; 60 of them did competitive sports (86%), and only 10 patients did recreational sports (14%). There was no statistically significant difference in the proportion of competitive and recreational sports within the revision group (90%) and the contralateral ACL reconstruction group (85%). (revision: *P*= 0.338; contralateral: *P*=0.236). (Table 3)

### Biological Sex

Eight of the athletes who underwent revision ACL reconstruction were female (80%), whereas the rate was even higher among those who underwent contralateral ACL reconstruction (females: 12 patients 92%; males: 1 patient 8%). The proportion of females was significantly greater in the case of contralateral ACL reconstruction (*P*=0.020), whereas for revision, the difference was not statistically significant (*P*=0.200). (Table 3)

### Comparison

The second group (Group 2) consisted of 143 patients who underwent ACL reconstruction between 2016 and 2020, all of whom were over 16 years old at the time of surgery. In this group, an online survey was conducted. Altogether, 86 athletes completed our questionnaire (60%). Compared with Group 1, the sex distribution ratio was the opposite (M53:F33). The mean age at ACL reconstruction was 30.8 (16.1–51.5 years) years, and the mean follow-up time was 3.66 (2.1–6.8 years) years. All 86 patients included completed the questionnaire in full.

In the second group (Group 2), 15 patients underwent any operation after primary ACL reconstruction (17%).

We found five revision ACL surgeries (5.8%) in Group 2. Among these five patients, three were under 20 years of age. The mean age of the five patients was 21.9 years. Among the procedures performed following ACL reconstruction, six arthroscopic arthrolysis procedures (6.9%), four meniscus resections (4.6%), and one (1%) meniscal refixation, microfracture, and metal removal procedure were performed. Furthermore, 11 patients underwent multiple operations on the ipsilateral side (12%).

On the contralateral side, four patients (4.6%) required surgery: one ACL reconstruction (1%), two meniscus resections (2.3%) and one meniscus refixation (1%).

The average age of the two groups showed a significant difference (Group1: 14,8 years vs. Group2: 30,8 years; *P*<0.0001).

A comparison of the groups is shown in Table 4.

**Table 4:** Comparison of the groups

Groups	Group1 (x<16)	Group2 (x>16)
Number	45	86
follow-up (x<8years)	3.72	3.66
Sex (F/M)	33:12	33:53
Age (Years)	14.8	30.8
Tegner before	8.0	7.8

Legend: This table summarizes the baseline characteristics of the two study groups based on age: Group 1 includes patients younger than 16 years, and Group 2 includes patients older than 16 years at the time of surgery

The table shows the number of patients in each group, mean follow-up time (in years), sex distribution (female:male), mean age, and the average Tegner activity level before injury

The number of meniscus surgeries (Group 1: 26% vs. Group 2: 4.6%; *P*=0.003), contralateral ACL reconstruction (Group 1: 8.8% vs. Group 2: 1.1%; *P*=0.03) and total number of surgeries (Group 1: 44% vs. Group 2: 21%; *P*=0.01) were significantly greater among patients under 16 years of age. We also found several cases of revision ACL reconstruction in Group 1, although the difference was no significant (Group 1: 11% vs. Group 2: 5.8%; *P*=0.293). (Table 5)

**Table 5:** Comparison of the groups based on the number of surgeries

Groups	Group1 (x<16)	Group2 (x>16)	
Revision ACL reconstruction	5/45 (11%)	5/86 (5.8%)	*P*=0.293
Meniscus surgery	12/45 (26%)	4/86 (4.6%)	*P*=0.003
Contralateral ACL reconstruction	4/45 (8.8%)	1/86 (1.1%)	*P*=0.03
Total number of surgeries	20/45 (44%)	18/86 (21%)	*P*=0.01

Legend: This table compares the surgical outcomes between Group 1 (patients under 16 years) and Group 2 (patients over 16 years), such as revision ACL reconstruction (*p* = 0.293), meniscus surgery (*p* = 0.003), contralateral ACL reconstruction (*p* = 0.03), and total number of surgeries (*p* = 0.01), with statistically significant differences observed in all except revision ACL reconstruction

The Lysholm scores (Group 1: 88.3 vs. Group 2: 87.0; *P*=0.271), IKDC subjective scores (Group 1: 88.4 vs. Group 2: 86.9; *P*=0.478), mean KOOS scores (Group 1: 86.9 vs. Group 2: 86.5; *P*=0.498), KOOS QOL scores (Group 1: 75.4 vs. Group 2: 67.4; *P*=0.064), daily scores (Group 1: 98.6 vs. Group 2: 98.8; *P*=0.453), and sport scores (Group 1: 89.6 vs. Group 2: 87.3; *P*=0.324) did not significantly differ, whereas the KOOS symptom scores (Group 1: 75.6 vs. Group 2: 85.0; *P*<0.005) were significantly lower in Group 1. However, the KOOS pain subscale score was significantly lower in patients over 16 years of age (Group 1: 95.6 vs. Group 2: 94.0; *P*=0.033). (Table 6)

**Table 6:** Comparison of the groups on the basis of patient-reported outcomes

	**Under 16 years**	**16 years and older**	
IKDC subj.	88.4	86.9	*P*=0.478
Lysholm	88.3	87.0	*P*=0.271
Mean KOOS	86.9	86.5	*P*=0.498
Pain	95.6	94.0	*P*=0.033
Sympt.	75.6	85.0	*P*<0.005
Daily	98.6	98.8	*P*=0.453
Sport	89.6	87.3	*P*=0.324
QOL	75.4	67.4	*P*=0.064

Legend: Comparison of postoperative outcome scores between patients under 16 and those 16 and older. Statistically significant differences were found in KOOS Pain (*P*=0.033) and KOOS Symptoms (*P*<0.005)

## Discussion

The most important findings of our study were that the rates of contralateral ACL reconstruction (*P*=0.03), meniscal surgery (*P*=0.003), and the total number of operations (*P*=0.01) were significantly higher in patients under 16 years of age compared to those over 16 years. Although ACL revision rates also appeared higher in the younger group, this difference did not reach statistical significance (*P*=0.293).

The question arises whether the lower contralateral rupture and re-rupture rates in the patients over 16 years could be due to the shorter follow-up period. As the years progress, the occurrence of repeat injuries naturally increases, but we do observe that re-injuries are most common in the first 2–3 years following ACL reconstruction [13, 23]. In our own study, graft re-rupture occurred on average 28 months after ACL reconstruction. Among the 13 cases of contralateral ACL rupture, the injury occurred within 3.6 years after ACL reconstruction in 6 cases, and later than 3.6 years in 7 cases. Given that the vast majority of re-ruptures occur within the first 36 months, we would have observed a higher rate in Group 2 even within the shorter 44-month follow-up period. We believe that the shorter follow-up period does not have a significant impact on the higher rate of revision injuries; however, to eliminate this potential source of bias, we standardized the follow-up period.

During the last three decades, there has been a high increase in the incidence of adolescent ACL rupture [24]. The increased number of young athletes may be responsible for the increased number of injuries, but early sports specialization and increased awareness of these injuries among the public and physicians may also play a role [3, 25]. One other possible reason for the higher rate of ACL graft re-rupture observed in young individuals is the presence of generalized joint hypermobility. This condition is characterized by an inherently more lax ligamentous system, which provides reduced joint stability and may increase the risk of graft failure under physical stress [26]. Return to sport is a well-known risk factor, and an early return to pivoting sports in particular significantly increases the risk of reinjury [27]. Compared with adult patients (83%), skeletally immature athletes have a greater return to sport level (92%), which may further increase the risk of graft re-rupture [28–31].

In our experience, the number of ACL reconstructions has steadily increased between 2000 (5 operations/year) and 2019 (23 operations/year), reflecting a growing trend in surgical treatment among adolescents.

Two well-known and important risk factors for ACL rupture are previous ACL reconstruction, therefore, it is legitimate to question whether we should perform the reconstruction in the case of an anterior cruciate ligament tear [15]. When an adult patient does not experience any symptoms or a locking sensation and does not participate in pivoting sports, conservative treatment may be considered an option [32]. In contrast, nonoperative ACL rupture care has explicitly negative outcomes among adolescents; therefore, we should aim for the earliest possible surgical treatment [10, 11, 33–35]. Nevertheless, not only are the revision rates among adolescents high, with rates ranging from 9–35% but also the contralateral ACL reconstruction rates (13–20%) are high [12, 16, 17, 36, 37, 39, 40]. The surgical results may be affected by the method of graft fixation, which is why Calvo's study is interesting, as he reported similar revision ACL reconstruction rates with the same surgical technique [41]. Calvo et al. used the EndoButton suspensory system with double-staple tibial fixation and reported more than 5 mm tibial translation in 22% of cases with respect to the healthy knee, although there was no statistically significant difference in functional scores between these patients and those with a difference of <5 mm. However, in our study, the rate of tibia translation greater than 5 mm was only 2.8%. Despite the difference in tibial translation, the revision rate was nearly the same (our study: 14.5% vs Calvo: 14,8%).

The double press-fit fixation technique used in patellar tendon reconstruction is relatively uncommon. Pavlik and colleagues investigated the stability of press-fit fixation and found that, at a 60-degree femoral tunnel angle, graft rupture or bony block fracture was more likely to occur. In contrast, at a 15-degree fixation angle, the overall stability was significantly reduced. Compared to data in the literature, these results demonstrated similar and satisfactory biomechanical properties of femoral press-fit fixation [42]. In Hidas’s study, histological analysis revealed adequate ossification by six weeks following the press-fit technique [43]. A major advantage of double press-fit fixation is that, in addition to providing satisfactory clinical outcomes, it eliminates the need for implant placement [22]. As a result, the bony block achieves more than 90% integration on the femoral side, which significantly facilitates the placement of the femoral tunnel in potential revision surgeries [44].

In our sample, 28 patients (39.4%) required meniscus surgery following ACL reconstruction after 6.9 years of follow-up. At a follow-up of 3.66 years, the rate of meniscus injuries was lower, at only 26%, indicating that the rate of meniscus injuries increased over time. However, patients older than 16 years had a significantly lower rate of meniscal tears (*P*=0.003). Fewer data are available on the rate of meniscal tears following ACL reconstruction than on revision rates. Chan's study reported that 10.7% of meniscal tears occurred after ACL reconstruction, whereas Ichinohe reported that only 4% of cases occurred [45, 46]. Unfortunately, in our study, we found a higher rate in patients under 16 years of age than in those under 16 years of age at both 3.66 and 6.9 years of average follow-up. For patients older than 16 years, the rate was 4.6%, which is in line with the literature.

Among patients who have been followed up, there are twice as many females as males (females: 48, males: 22). Nicholls et al. reported that among the adolescent population, ACL ruptures affect females the most [47]. Hypermobility syndrome (HMS) is a predictive factor for revision and contralateral ACL surgeries and is also more common among females [48]. This may be due to the high number of females in both our groups (revision: 80%, contralateral: 92%). The proportion of females was significantly greater in the case of contralateral ACL reconstruction (*P*=0.020), whereas for revision, the difference was not statistically significant (*P*=0.200). Ahldén et al. also reported that young female athletes have a significantly greater incidence of contralateral ALC ruptures when analyzing data from the Swedish Ligament Register [19].

Wiggins et al. reported that returning to sports increases the rate of reinjury [12]. Moreover, it also makes a difference in which sport the athlete returns to. Athletes who play high-pivoting sports (e.g., football) have a greater risk of ACL rerupture [38, 49]. In contrast to ice hockey and basketball, Boch reported higher rates of reinjury among handball and football players [8]. We hypothesized that the rates of revision and contralateral ACL reconstruction would be greater among competitive athletes performing high-pivoting ball sports. We could not prove our theory. The rates of revision and contralateral cruciate ligament reconstruction were not significantly higher among either competitive (revision: recreational (10%) vs. competitive (15%), *P*=0,337); contralateral (recreational (11%) vs. competitive (18%), *P*=0,236) or pivoting athletes (revision: other (11%) vs. HSB (15%), *P*=0,326); contralateral (other (11%) vs. HSB (21%), *P*=0,173)).

There were no significant differences in patient-reported outcomes, except for the KOOS symptoms score (*P* < 0.005). Surprisingly, the KOOS pain subscale score was significantly higher in the under 16-year-old athletes (*P*=0.033).

Our study supports the conclusion that ACL reconstruction performed during adolescence yields less favorable surgical outcomes compared to procedures carried out at an older age, particularly in terms of re-rupture rates and contralateral ACL rupture. Reducing this unfavorable outcome is of paramount importance for orthopedic surgeons, as minimizing re-rupture and contralateral ACL rupture rates is essential to improving long-term functional results in adolescent patients. One of the most promising techniques of the last decade is lateral extra-articular tenodesis (LET). This procedure has shown significant benefits in improving outcomes in ACL reconstruction, particularly for young, high-activity patients. It helps stabilize the knee, reducing re-rupture and graft failure rates among individuals with high sports activity [50–54].

### Limitations

This retrospective study has several limitations. First, we did not use a questionnaire before the ACL reconstruction, so we could not measure clinical outcomes.

Second, the follow-up time varied widely (mean 6.9; 2–19 years), so in our study, the follow-up duration was only medium-term. To compare the groups, the follow-up time of the first group had to be reduced, which unfortunately reduced the number of cases as well. Another limitation of the medium-term follow-up is that any subsequent surgeries or re-injuries that may occur later are not documented.

Because of administrative issues, 194 patients were lost to follow-up out of our 289 patients. Following the request, 70 patients were personally involved in our study (36%). Unfortunately, this rate is low; however, Ahldén et al. reported a completion rate of approximately 40% in the Swedish cruciate ligament register at the 5-year follow-up [19].

Another limitation of the study is that we did not analyze the return to different sports or the level of return separately. The level of post-surgery sports activity was only addressed in terms of examining which sports were practiced by patients who underwent revision surgery or contralateral ACL reconstruction, as well as in the comparison of Tegner scores between the groups.

## Conclusion

In our study, the total number of surgeries, particularly meniscal procedures was significantly higher among athletes under the age of 16. Revision and contralateral ACL reconstructions were also more frequent in this age group; however, these differences did not reach statistical significance. A significantly higher proportion of females underwent contralateral ACL reconstruction. These findings highlight age and sex as potential factors associated with surgical outcomes following ACL injuries.

## Data Availability

No datasets were generated or analysed during the current study.

## Declarations

All procedures performed in our studies involving human participants were in accordance with the ethical standards of the institutional and national research committees and with the 1964 Helsinki Declaration and its later amendments or comparable ethical standards. The study was approved by the regional ethical committee (No. 15/2019).

The datasets used and analyzed during the current study are available from the corresponding author upon reasonable request.
